# One-Legged Balance Performance and Fall Risk in Mid and Later Life: Longitudinal Evidence From a British Birth Cohort

**DOI:** 10.1016/j.amepre.2022.07.002

**Published:** 2022-12

**Authors:** Joanna M. Blodgett, Rebecca Hardy, Daniel Davis, Geeske Peeters, Diana Kuh, Rachel Cooper

**Affiliations:** 1 Division of Surgery & Interventional Science, Institute of Sport, Exercise & Health, University College London, London, United Kingdom; 2 MRC Unit for Lifelong Health and Ageing at UCL, UCL Institute of Cardiovascular Science, London, United Kingdom; 3 Cohort and Longitudinal Studies Enhancement Resources, Social Research Institute, University College London, London, United Kingdom; 4 Department of Geriatric Medicine, Radboud University Medical Centre, Nijmegen, The Netherlands; 5 Department of Sport and Exercise Sciences, Musculoskeletal Science and Sports Medicine Research Centre, Manchester Metropolitan University Institute of Sport, Manchester, United Kingdom; 6 AGE Research Group, NIHR Newcastle Biomedical Research Centre, Translational and Clinical Research Institute, Newcastle University, Newcastle upon Tyne, United Kingdom; 7 NIHR Newcastle Biomedical Research Centre, Newcastle University and Newcastle upon Tyne Hospitals NHS Foundation Trust, Newcastle upon Tyne, United Kingdom

## Abstract

**Introduction:**

The one-legged balance test is widely used as a fall risk screening tool in both clinical and research settings. Despite rising fall prevalence in midlife, there is little evidence examining balance and fall risk in those aged <65 years. This study investigated the longitudinal associations between one-legged balance and the number of falls between ages 53 and 68 years.

**Methods:**

The study included 2,046 individuals from the Medical Research Council National Survey of Health & Development, a British birth cohort study. One-legged balance times (eyes open, maximum: 30 seconds) were assessed at ages 53 years (1999) and 60–64 years (2006–2010). Fall history within the last year (none, 1, ≥2) was self-reported at ages 60–64 years and 68 years (2014). Multinomial logistic regressions assessed the associations between balance and change in balance with subsequent falls. Models adjusted for anthropometric, socioeconomic, behavioral, health status, and cognitive indicators. Analysis occurred between 2019 and 2022.

**Results:**

Balance performance was not associated with single falls. Better balance performance at age 53 years was associated with decreased risk of recurrent falls at ages 60–64 years and 68 years, with similar associations between balance at age 60–64 years and recurrent falls at age 68 years. Those with consistently lower balance times (<15 seconds) were at greater risk (RRR=3.33, 95% CI=1.91, 5.80) of recurrent falls at age 68 years in adjusted models than those who could balance for 30 seconds at ages 53 years and 60–64 years.

**Conclusions:**

Lower balance and consistently low or declining performance were associated with a greater subsequent risk of recurrent falls. Earlier identification and intervention of those with poor balance ability can help to minimize the risk of recurrent falls in aging adults.

## INTRODUCTION

The WHO estimates that a third of individuals aged ≥65 years and half of those aged ≥80 years fall each year.^1^^,^^2^ Falls can restrict mobility, can decrease independence, and are the leading cause of injury-related death in older adults.^1^^‒^^3^ Secular trends suggest that the prevalence of fall-related injuries,^4^^,^^5^ hospitalization,^5^, ^6^, ^7^, ^8^ and mortality^9^, ^10^, ^11^ has increased over time. These increases persist in age-adjusted analyses, indicating that this rise is not attributable to population aging alone.^12^^,^^13^

Pooled data from Great Britain, Ireland, Australia, and The Netherlands indicate that fall prevalence rises during midlife from 8.7% (age 43 years) to 20.9% (age 50–54 years) to 29.9% (age 60–64 years) in women, with similar increases in men.^14^ Despite meaningful fall prevalence in midlife, there is minimal evidence examining the risk factors for falls in middle-aged adults.^14^, ^15^, ^16^ Furthermore, functional declines have been observed up to 12 years before an individual's first fall,^17^ suggesting that midlife may provide an extensive window for preventive measures if at-risk individuals can be identified.

Many research and clinical efforts have focused on balance ability as an indicator of future fall risk.^18^ A common balance assessment is the one-legged stand, which is widely adopted in research and clinical settings because of its low cost and implementation burden,^19^, ^20^, ^21^ high inter-rater and test‒retest reliability,^22^, ^23^, ^24^, ^25^, ^26^, ^27^ and strong concurrent and predictive validity.^20^^,^^25^^,^^28^, ^29^, ^30^, ^31^, ^32^ However, a recent systematic review of 55 papers examining one-legged balance and falls reported low quality of evidence.^33^ Most studies (60%) measured balance performance and self-reported fall history at the same time; the likelihood of reverse causality (e.g., fall history leading to poor balance) in cross-sectional analyses is high. Despite single fallers being more similar to nonfallers than to recurrent fallers,^31^^,^^34^^,^^35^ 69% of studies assessed falls as a binary (0 vs ≥1) outcome. Crucially, studies rarely adjusted for confounders nor examined how associations differed by age or sex, despite clear differences in balance ability and fall prevalence for males and females of different ages.^36^, ^37^, ^38^, ^39^

There is a vital need to address these limitations and understand whether one-legged balance is associated with fall risk in midlife using large, longitudinal population-representative studies. This study aimed to examine the longitudinal associations of one-legged balance performance and change in balance with fall frequency at ages 60–64 years and 68 years in a large, representative British cohort study. Secondary objectives were to investigate whether the associations differed by sex, changed with age, and were robust to adjustment for fall-related covariates.

## METHODS

This study follows the STROBE guidelines.^40^

### Study Sample

**Figure 1:**
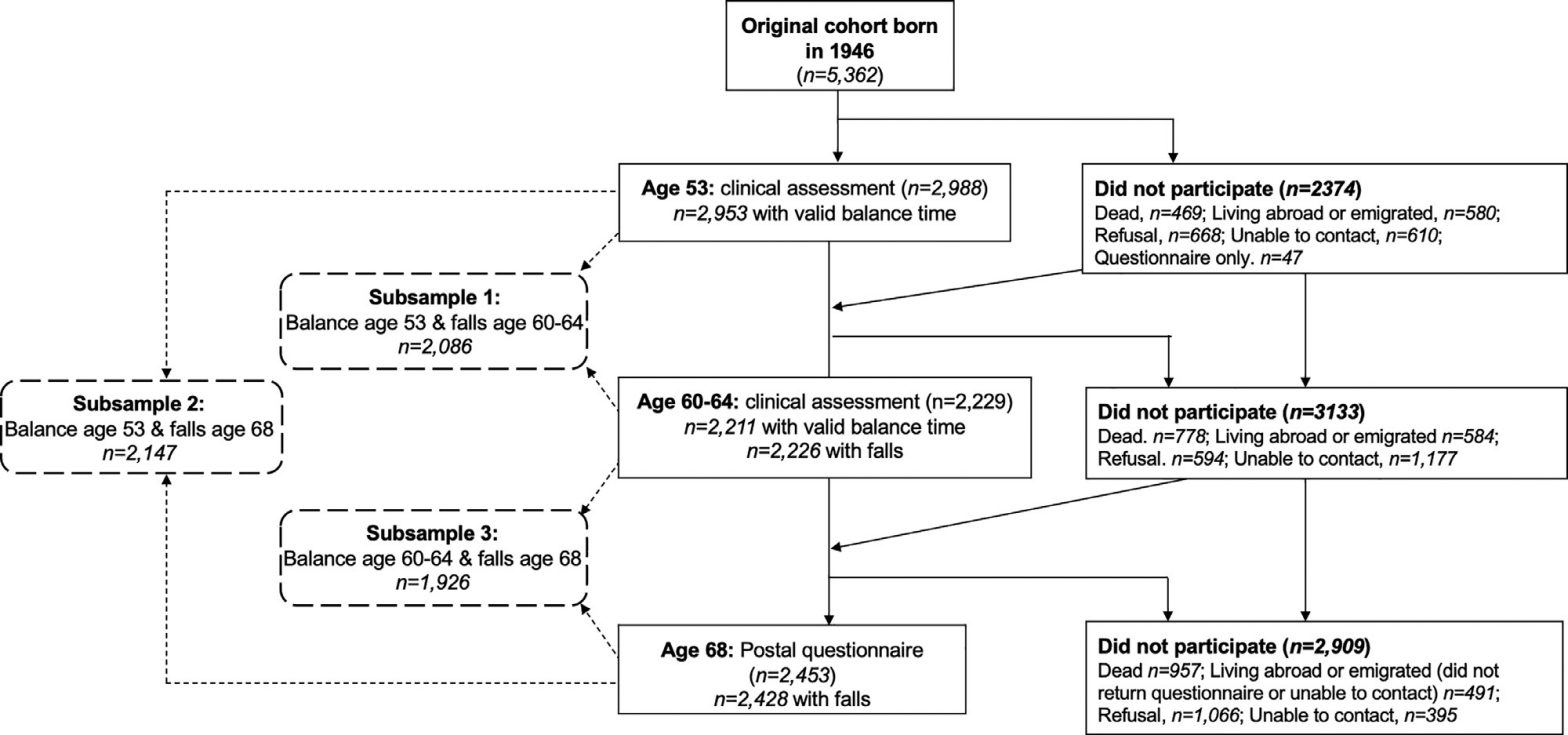
Flow diagram denoting participation in data collections at ages 53 years, 60–64 years, and 68 years and the derivation of the 3 analytical subsamples. *Note*: Black lines indicate the flow of subject members from birth to age 68 years; dotted lines indicate derivation of each subsample.

The Medical Research Council National Survey of Health and Development is an ongoing birth cohort study of 5,362 individuals born in England, Scotland, or Wales during 1 week in March 1946. Individuals have been followed since birth, providing prospectively ascertained data at up to 24 time points. Reasons for nonparticipation have been described previously.^41^, ^42^, ^43^ Balance performance and fall history were assessed at ages 53 years (1999), 60–64 years (2006–2010), and 68 years (2014). At ages 53 years and 60–64 years, 2,988 and 2,229 individuals, respectively, participated in clinical assessments; at age 68 years, 2,453 returned a postal questionnaire. Participants were included in the analytical sample if they had balance data at age 53 years or 60–64 years and falls data at a subsequent age. Because sample size differed across the 3 waves, 3 subsamples maximized sample size (Figure 1); a total of 2,496 individuals were included across all subsamples. The most recent ethical approval was provided by the Queen Square Research Ethics Committee (13/LO/1073) and Scotland A Research Ethics Committee (14/SS/1009). Data analysis occurred between 2019 and 2022.

### Measures

Participants were instructed to cross their arms and lift their preferred leg a few inches off the ground. Nurses recorded the time the participant could maintain the position to the nearest second (age 53 years) or 1/100th of a second (age 60–64 years). Time stopped when the participant's raised foot touched the ground or after 30 seconds. Participants performed 1 eye open and 1 eye closed trial; eyes open times were used in these analyses because they are the most common visual condition for this test.^33^ Individuals who were unable to perform the test because of health reasons were allocated a balance time of 0 seconds (*n*=61 at age 53 years only, *n*=75 at age 60–64 years only, *n*=14 at both ages).^44^

A total of 5 groups indicated a change in balance performance from age 53 years to 60–64 years: stable high (30 seconds at both ages), stable middle (15 to <30 seconds), stable low (0 to <15 seconds), and improved and declined (moving from better to worse categories between ages 53 years and 60–64 years, respectively). A total of 15 seconds was chosen as the cut off for the change categories because of strong ceiling effects (69% and 50% balanced for 30 seconds at ages 53 years and 60–64 years, respectively) and to ensure a sufficient sample size across the other categories.

At age 60–64 years, participants reported whether they had fallen at all in the past 12 months during interviews. At age 68 years, individuals were asked through postal questionnaire *in the past 12 months [if they] had any fall including a slip or trip in which [they] lost [their] balance and landed on the floor or ground or lower level*.^45^ Number of falls were categorized as none, single, or recurrent (≥2).

Covariates for ages 53 years and 60–64 years were identified a priori from previous research on the basis of associations with falls.^44^^,^^46^ The anthropometric measures of height (cm) and weight (kg) were ascertained using standard protocols by research nurses and used to calculate BMI (kg/m^2^). Socioeconomic position was evaluated using the highest level of educational attainment up to age 26 years (categorized as degree or higher, advanced secondary qualifications typically attained at age 18 years, ordinary secondary qualifications typically attained at age 16 years, below ordinary secondary qualifications, or none) and occupational class, which was derived from self-reported occupation at age 53 years and categorized using the Registrar General's Social Classification (I, professional/II, intermediate; III, skilled nonmanual/manual; IV, partly skilled/V, unskilled manual).^47^

Self-reported health behaviors included leisure-time physical activity, which was categorized as never, 1–4 times/month, or ≥5 times/month, and smoking status, which was ascertained using data from current and previous waves (never, ex-smoker, current smoker). Health status‒related indicators included self-reported measures of knee pain, diabetes history, previous cardiovascular event, respiratory symptoms, fall history (all yes/no), depression and anxiety symptoms (28-item General Health Questionnaire, range=0–84), and medication count (continuous). Cognition was examined using verbal memory, which was assessed using 3, 15-item word learning task trials. Each word was presented for 2 seconds; participants were instructed to write down all the words they could remember (maximum score:45).

### Statistical Analysis

Differences in covariates between nonfallers, single fallers, and recurrent fallers at age 60–64 years were assessed using chi-square tests, 1-way ANOVA, and Kruskal‒Wallis tests with posthoc Bonferroni or Dunn's tests, as appropriate. Multinomial logistic regression models were used to assess the associations between balance performance and falls (0, 1, ≥2) as follows: (1) balance age 53 years and falls age 60–64 years, (2) balance age 53 years and falls age 68 years, (3) balance age 60–64 years and falls age 68 years, and (4) change in balance (age 53 years to 60–64 years) and falls age 68 years. For each temporal association, interactions between sex and balance were assessed. Five models were considered with adjustment in turn for anthropometric measures, socioeconomic position, health behaviors, health status‒related indicators, and cognition; a final model included all covariates. The reference category for all models was no falls, and all estimates are presented as RRRs.

Missing covariate data were imputed using multiple imputation by chained equations under a missing-at-random assumption. Rubin's rules were used to combine estimates across the 20 imputed data sets.^48^ Father's occupational class (age 4 years) and serious childhood illness (≥28 days of hospital admission before age 25 years) are strong predictors of missingness in British birth cohort studies^49^ and therefore were included as auxiliary variables to improve imputation accuracy. Missing data ranged from 0% (several variables) to 6.3% (smoking status at age 60–64 years). The following sensitivity analyses were used to quantify the robustness of the models: excluding those missing covariates, excluding those who could not complete balance tests because of health problems, and excluding those who fell within the 12 months before balance assessments. Characteristics of complete cases, those missing covariates, and those lost to follow-up were also compared. Statistical analyses were conducted in Stata 17.

## RESULTS

Single and recurrent fallers were more commonly female, shorter, less active, and had higher educational attainment than nonfallers and were more likely to have poor physical and mental health and greater medication use (Table 1). Bonferroni posthoc tests suggested minimal differences between nonfallers and single fallers, several differences between single and recurrent fallers, and the largest differences between nonfallers and recurrent fallers.

**Table 1:** Descriptive Characteristics of Maximal Analytical Sample (Up to N=2,496) by Fall Status at Age 60–64 Years

Variables	0 falls (*n*=1,787; 81.7%)	1 fall (*n*=226; 10.3%)	≥2 falls (*n*=173; 7.9%)	*p*-value
Sex, *n* (%)				<0.01
Male	902 (50.5)	84 (37.2)	55 (31.6)	
Female	885 (49.5)	142 (62.8)	118 (68.5)	
Anthropometry at age 60–64 years, mean ± SD				
Height (m)	1.68 ± 0.09	1.67 ± 0.09	1.66 ± 0.08	<0.001
BMI (kg/m^2^)	27.9 ± 4.9	27.7 ± 4.6	28.8 ± 5.5	0.06
Socioeconomic indicators, *n* (%)				
Educational attainment up to age 26 years				
Degree or higher	204 (12.0)	22 (10.1)	9 (5.7)	0.03
A levels, usually attained at age 18 years	479 (28.2)	66 (30.6)	39 (24.7)	
O levels, usually attained at age 16 years	341 (20.1)	55 (25.5)	44 (27.9)	
Secondary education or clerical course	121 (7.1)	18 (8.3)	15 (9.5)	
None attempted	553 (32.6)	55 (25.5)	51 (32.3)	
Highest occupational class at age 53 years				
I, professional/II, intermediate	830 (46.6)	112 (50.0)	72 (43.1)	0.23
III, skilled (nonmanual or manual)	708 (39.8)	85 (37.6)	62 (37.1)	
IV, partly skilled/V unskilled	243 (13.6)	29 (12.8)	33 (19.8)	
Behavioral risk factors at age 60–64 years, *n* (%)				
Leisure-time physical activity				
None	1,107 (63.8)	127 (57.5)	116 (72.1)	0.03
1–4 times/month	237 (13.7)	41 (18.6)	14 (8.7)	
≥5 times/month	391 (22.5)	53 (24.0)	31 (19.3)	
Smoking status				
Current	182 (11.1)	24 (11.3)	16 (10.8)	0.62
Previous smoker	925 (56.5)	113 (53.3)	91 (61.5)	
Never smoker	530 (32.4)	75 (35.4)	41 (27.7)	
Health-status related indicators at age 60–64 years				
History of diabetes, *n* (%)	145 (8.1)	13 (5.8)	17 (10.1)	0.27
History of CVD events, *n* (%)	113 (7.2)	18 (8.7)	18 (12.6)	0.06
Respiratory symptoms, *n* (%)	263 (16.7)	47 (22.4)	46 (32.4)	<0.001
Knee pain, *n* (%)	359 (20.2)	51 (22.6)	77 (45.8)	<0.001
Any previous fall history, *n* (%)	231 (13.6)	49 (22.4)	63 (39.9)	<0.001
Symptoms of anxiety/ depression, mean (SD)	15.9 (7.7)	17.3 (8.6)	21.5 (10.6)	<0.001
Medications, median (Q1, Q3)	2 (0,4)	2 (0,4)	3 (1,5)	<0.001
Cognition at age 60–64 years, *n* (%)				
Verbal memory, mean ± SD	24.5 ± 6.1	25.4 ± 6.1	24.0 ± 6.6	0.08

^a^*p*-values indicate differences between nonfallers, single fallers, and recurrent fallers using chi-square or 1-way ANOVAs.

CVD, cardiovascular disease.; Q, quartile.

**Figure 2:**
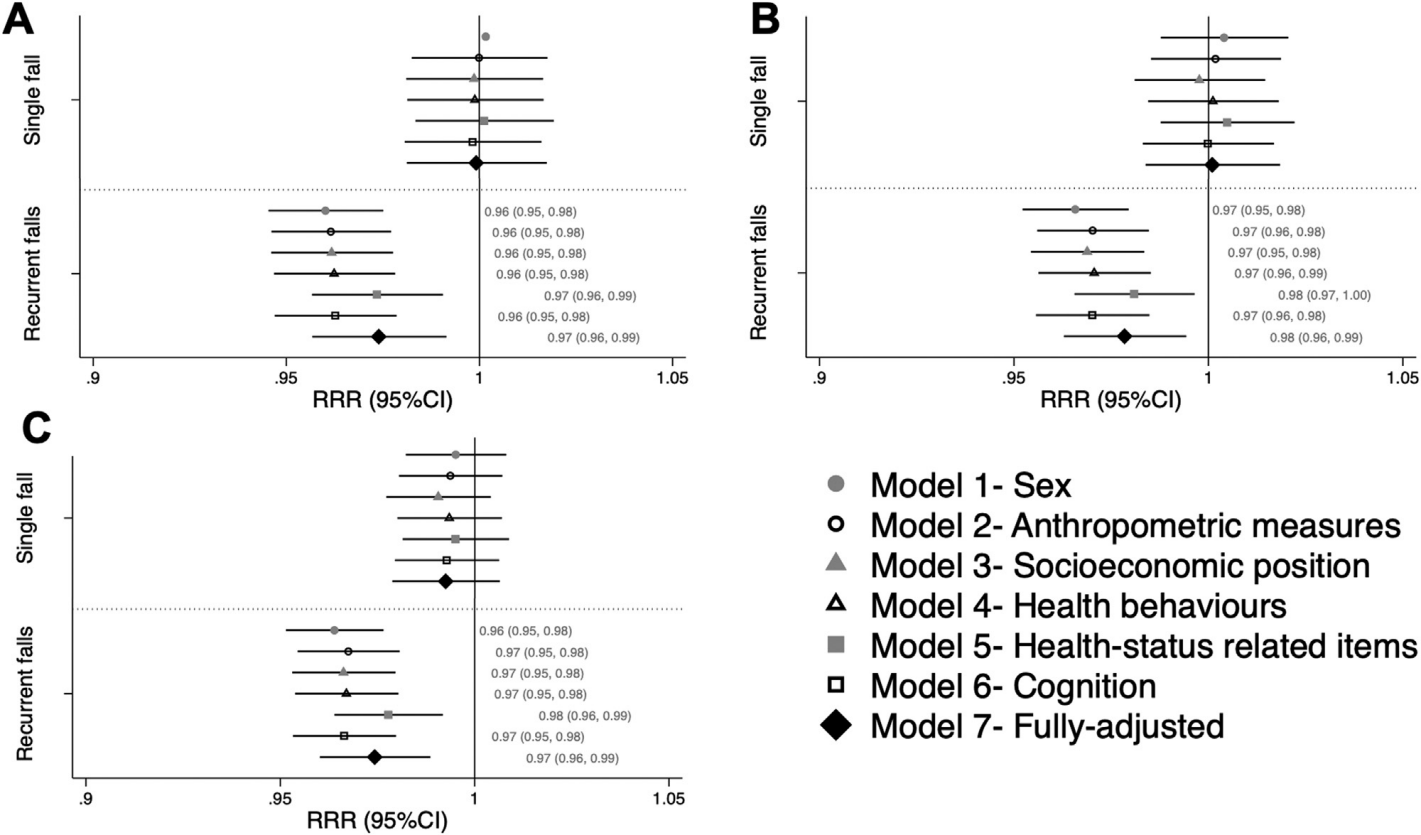
RRRs per 1-second increase in balance with eyes open at age (A) 53 years and falls at age 60–64 years, (B) 53 years and falls at age 68 years, and (C) 60–64 years and falls at age 68 years. *Note*: Estimates >1 suggest an increased fall risk, whereas estimates <1 suggest a reduced fall risk; statistical significance is indicated if 95% CIs do not cross 1.

There were no sex‒balance interactions; therefore, males and females were considered together. There were no associations between balance performance and single fall risk at any age (Figure 2A‒C). Better balance performance at age 53 years was associated with lower RR of recurrent falls (versus no falls) at ages 60–64 years (RRR sex-adjusted model=0.96 95% CI=0.95, 0.98 per 1 second increase in balance) (Figure 2A) and at 68 years (RRR sex-adjusted model=0.97; 95% CI=0.95, 0.98) (Figure 2B). Similarly, better balance performance at age 60–64 years was associated with a lower RR of recurrent falls at age 68 years (RRR=0.96; 95% CI=0.95, 0.98) (Figure 2C). An RR of 0.96 indicates a reduction in the RR of recurrent falls of 4% (versus no falls) for each additional second an individual maintained their balance. Adjustment for anthropometric, socioeconomic, behavioral, health, and cognitive factors did not explain the associations. At all ages, adjustment for health status‒related indicators attenuated the estimates the most; however, no single indicator fully explained this attenuation (Appendix Figure 1, available online).

Of 2,496 individuals, 1,827 (73%) had balance data at ages 53 years and 60–64 years and self-reported falls at age 68 years. These individuals had comparable fall prevalence at ages 60–64 years and 68 years (*p*>0.70) but better balance performance at ages 53 years (median: 30 seconds [quartile (Q)1:27, Q3:30] vs 30 seconds [20, 30]) and 60–64 years (30 seconds [11.43, 30] vs 22.8 seconds [8.0, 30]) than those not included (*n*=669, 26.8%). Most individuals had a stable high balance (44.9%), 34.9% declined from age 53 years to 60–64 years, 8.6% improved, 7.7% had a stable low balance, and 3.8% maintained a stable middle balance.

There was no association between change in balance from age 53 years to 60–64 years and single fall risk at age 68 years (Appendix Table 1, available online). All groups had a greater risk of recurrent falls (than no falls) than those with stable high balance (Table 2). For example, those with stable low balance had the highest risk (RRR=4.24; 95% CI=2.58, 6.96), followed by those with stable middle (RRR=2.97; 95% CI=1.49, 5.92) or declining (RRR=1.75; 95% CI=1.20, 2.56) balance. An RRR of 4.24 indicates a 324% increase in the risk of recurrent falls (versus no falls) for those with a stable low balance, compared with that for those with a stable high balance. Estimates were robust to adjustment.

**Table 2:** RRRs Indicating the Associations Between Change in Balance From Age 53 Years to 60–64 Years and Recurrent Falls at Age 68 Years

Groups	Defined	Sex-adjusted model	*p*-value	Fully-adjusted^a^	*p*-value
Stable high (*n*=821; 44.9%)	Achieved 30 seconds at ages 53 years and 60–64 years	Reference group		Reference group	
Improved (*n*=157; 8.6%)	Improved from 0 to 14.99 seconds to 15–30 seconds or from 15 to 29.99 seconds to 30 seconds	1.72 (0.97, 3.08)	0.07	1.66 (0.91, 3.03)	0.10
Declined (*n*=638; 34.9%)	Declined from 30 seconds to 0–29.99 seconds or from 15 to 29.99 seconds to 0–14.99 seconds	1.75 (1.20, 2.56)	<0.005	1.74 (1.17, 2.59)	<0.01
Stable middle (*n*=70; 3.8%)	Recorded 15–29.99 seconds at both ages	2.97 (1.49, 5.92)	<0.005	2.86 (1.39, 5.91)	<0.005
Stable low (*n*=141; 7.7%)	Stayed in 0–14.99 seconds group	4.24 (2.58, 6.96)	<0.001	3.33 (1.91, 5.80)	<0.001

*Note:* Estimates >1 suggest an increased fall risk, whereas estimates <1 suggest a reduced fall risk; statistical significance is indicated if 95% CIs do not cross 1. Associations between change in balance and single falls were not statistically significant for any model (*p*>0.25) (Appendix Table 1, available online, provides estimates).

a Model adjusted for anthropometric measures (BMI, height), socioeconomic position (educational attainment, occupational class), health behaviors (leisure-time physical activity, smoking status), health status‒related indicators (knee pain, history of diabetes, history of cardiovascular events, respiratory symptoms, history of falls, symptoms of depression and anxiety, medication count), and cognition.

Results did not change when analyses were restricted to complete cases (Appendix Figure 2, available online), those who completed the balance test (Appendix Figure 3, available online), or those with no fall history within the 12 months preceding balance assessment (Appendix Figure 4, available online). The analytical sample had higher occupational class (46.1% vs 31.7% professional/managerial), had higher educational attainment (38.6% vs 26.0% A level or higher), were more likely to be female (52% vs 43.6%), were less likely to be current smokers (11.4% vs 18.8%) or have a previous cardiovascular disease event (7.6% vs 13.2%), and had fewer anxiety and depression symptoms (16.5 ± 8.2 vs 19.6 ± 11.2) and better word recall (24.5 ± 6.2 vs 21.3 ± 6.2) than those excluded because of missing data or loss to follow-up.

## DISCUSSION

Better one-legged balance performance was associated with decreased risk of recurrent falls after up to 15 years of follow-up. Associations were observed from age 53 years to 68 years and were robust to adjustment for fall-related risk factors. There was no evidence of longitudinal associations between balance and single falls. When a change in balance performance was considered, those with consistently low or medium performance and those whose performance declined over time were at increased risk of recurrent falls compared with those who maintained high balance. This study directly addresses limitations from previous literature and shows novel insights, including associations in middle-aged adults, specific associations for recurrent compared with single falls, persistent associations over 15 years in midlife, and large associations between change in balance performance and subsequent fall risk.

Comparison between study results and current evidence is limited. Of 55 studies from the recent systematic review,^33^ 9 examined recurrent falls, and just 5 of these were longitudinal studies. All the 5 studies used cut-points to dichotomize balance performance; therefore, comparison of effect sizes is not possible. However, 3 of these 5 studies reported an unadjusted increased risk of recurrent falls in those with poor balance.^34^^,^^50^^,^^51^ Nevitt et al.^34^ was the only study to present findings from adjusted models (race, fall history, comorbidities, other physical performance tests), showing complete attenuation. Expanding to studies examining any fall outcome, 2 samples were of similar age to that of National Survey of Health and Development study participants (mean age: 55 years ± 22 years,^52^ 56.8 ± 4.4^53^); however, neither found an association.

Because there is limited recurrent falls evidence for comparisons, it is also valuable to compare results with those of another large, epidemiologic cohort study, which took a similar approach with injurious falls. The Swedish National Study on Ageing and Care in Kungsholmen is an age-stratified, random sample of a local neighborhood (*n*>2,000) that measured one-legged balance and investigated the risk of ICD-10 code‒derived injurious falls at multiple follow-up points (e.g., at 3, 4, 5, and 10 years; between 4 and 10 years).^54^, ^55^, ^56^ The authors stratified by sex and presented multiple models of adjustment (age, education, smoking, exercise, fall history, medications, comorbidities). Consistent with the study findings as mentioned earlier, associations remained, despite slight attenuation after adjustment and decreasing effect size with a greater length of follow-up.

Intrinsic factors, such as balance, play a dominant role in recurrent falls,^34^^,^^57^ whereas single occurrence falls may result from accidental extrinsic factors (e.g., environmental hazards). Therefore, it was unsurprising that no association between balance and single falls was observed. This is consistent with previous research identifying few risk factors associated with single falls and many associated with multiple falls.^34^^,^^58^ Furthermore, characteristics of those who fall 1 time are more similar to those of nonfallers than to those of recurrent fallers, as shown in this study and in previous literature.^31^^,^^34^^,^^35^ Adjustment for health status‒related items had the largest impact on attenuation. Further exploration of how specific comorbidities, medications (e.g., benzodiazepines, anti-hypertensives), psychological factors (e.g., fear of falling), fall history (e.g., 1 versus multiple), and other environmental factors may impact fall risk is needed. Considered alongside the evidence on injurious falls,^54^, ^55^, ^56^ individuals with poor one-legged standing balance ability may be more susceptible to recurrent or injurious falls. As the prevalence of falls in middle and older aged adults continues to increase, prevention efforts could improve efficiency by targeting balance interventions aiming to reduce high-risk falls.^59^ Assessment of one-legged balance ability earlier in midlife and at multiple time points provide 2 promising ways to identify individuals at the highest risk; further research is needed to translate these findings.

Previous evidence has highlighted the rising prevalence of falls during midlife.^14^ As shown in this study, associations of balance with falls risk clearly emerge before age 65 years, possibly because of declining mobility and physical function.^15^^,^^16^ Where possible, falls research in cohort and primary care settings should extend its scope to examine a wider age range, and fall prevention guidelines should include recommendations for those aged <65 years. These recommendations are strengthened by the substantially increased fall risk in those with persistently low or declining balance during midlife. The longevity of associations over a 15-year period suggests that there is a long window of opportunity for preventive interventions. Earlier understanding and screening of falls risk could enable appropriate interventions for individuals with the highest need.

Translation of observational associations into clinical implications must occur with caution. Explanatory associations (e.g., regressions) do not equate to predictive associations; this differentiation is crucial given the frequent use of one-legged balance as a screening tool in clinical settings.^19^^,^^20^ This study conducted exploratory modeling, often used to identify appropriate targets for intervention by understanding mechanistic pathways.^60^^,^^61^ The predictive ability of balance for fall risk must be formally evaluated using appropriate predictive modeling approaches.^62^ However, the findings highlight opportunities for preventive interventions in midlife and suggest that balance‒fall associations are independent of other fall-related factors. For example, several reviews have reported that balance training across life, including single leg training, can strongly improve balance ability.^63^, ^64^, ^65^, ^66^ Of note, the review of single-leg balance training suggested that benefits were seen after single sessions as short as 10 minutes.^63^

### Limitations

Self-reported fall measures are common in large-scale cohort studies, although accuracy is susceptible to recall bias, resulting in underestimation of fall prevalence. There were minor changes in the phrasing of the fall questions between waves; at age 68 years, wording from the ProFaNE guidelines was used,^45^ which may have improved the accuracy of reporting. The one-legged balance test is an isolated assessment of static balance, and the results may not be generalizable to dynamic balance. For example, static balance may have lower external validity in real-life settings, where dynamic balance is more commonly relied upon. Further comparison of static and dynamic tests in relation to fall risk is needed in studies that have assessed both measures. Individual balance performance could have been impacted by acute health conditions, psychological factors, or other extenuating circumstances; repeated balance assessments, as shown in this study, could provide a more accurate representation of balance ability. This is similar for fall risk because falls at each age were treated as independent events. Alongside balance or gait impairments, fall history is 1 of the 2 strongest predictors of future falls^67^; regular assessment of falls could inform longitudinal fall profiles and identify those at greatest cumulative risk. Variation in time between balance ascertainment in the age 60–64 years wave and falls ascertainment in the age 68 years wave may influence the current findings; this is unlikely to have had a major impact because most individuals were assessed at age 63 years or 64 years and thus had a follow-up time of 4–5 years. As highlighted in the results, those with missing data had poorer health behaviors, socioeconomic position, and cognitive and physical health, whereas loss to follow-up may have introduced bias.^41^^,^^42^ Although multiple imputation reduced the impact of missing data, attrition bias may limit the generalizability of findings. The predominant strength of this study is that it addressed most limitations identified in a recent systematic review examining the associations between one-legged balance performance and falls risk.^33^ This includes longitudinal follow-up of falls, a large population-representative sample, assessment of sex interactions, distinguishing between single and recurrent falls, inclusion of a wide range of covariates, and investigation of change in balance over time.

## CONCLUSIONS

In a large population-representative study, better one-legged balance performance was associated with a lower risk of recurrent falls in middle-aged adults. Associations were robust to adjustment for fall-related factors and were sustained for over 15 years of follow-up. The findings also showed that changes in balance performance in midlife are informative. This evidence directly addresses limitations in the current evidence and highlights the potential importance of one-legged balance performance at earlier ages for future risk of recurrent falls.

## Appendix SUPPLEMENTAL MATERIAL

Image, application 1

## Footnotes

Supplemental materials associated with this article can be found in the online version at https://doi.org/10.1016/j.amepre.2022.07.002.
