# Supplementary material for: One-Legged Balance Performance and Fall Risk in Mid and Later Life: Longitudinal Evidence From a British Birth Cohort
Source: Am J Prev Med. 2022 Dec;63(6):997–1006. doi: 10.1016/j.amepre.2022.07.002 (PMC10499759; doi:10.1016/j.amepre.2022.07.002)
Supplement: Supplementary file 1 [file mmc1.pdf]

Appendix Figure 1. Relative risk ratios, adjusted for individual health related indicators, per 1s increase in balance with eyes open at age A. 53 and recurrent falls at age 60-64 (n=2052); B. age 53 and recurrent falls at age 68 (n=2110); C. 60-64 and recurrent falls at age 68 (n=1920) [corresponding to Table 2, Model 5]

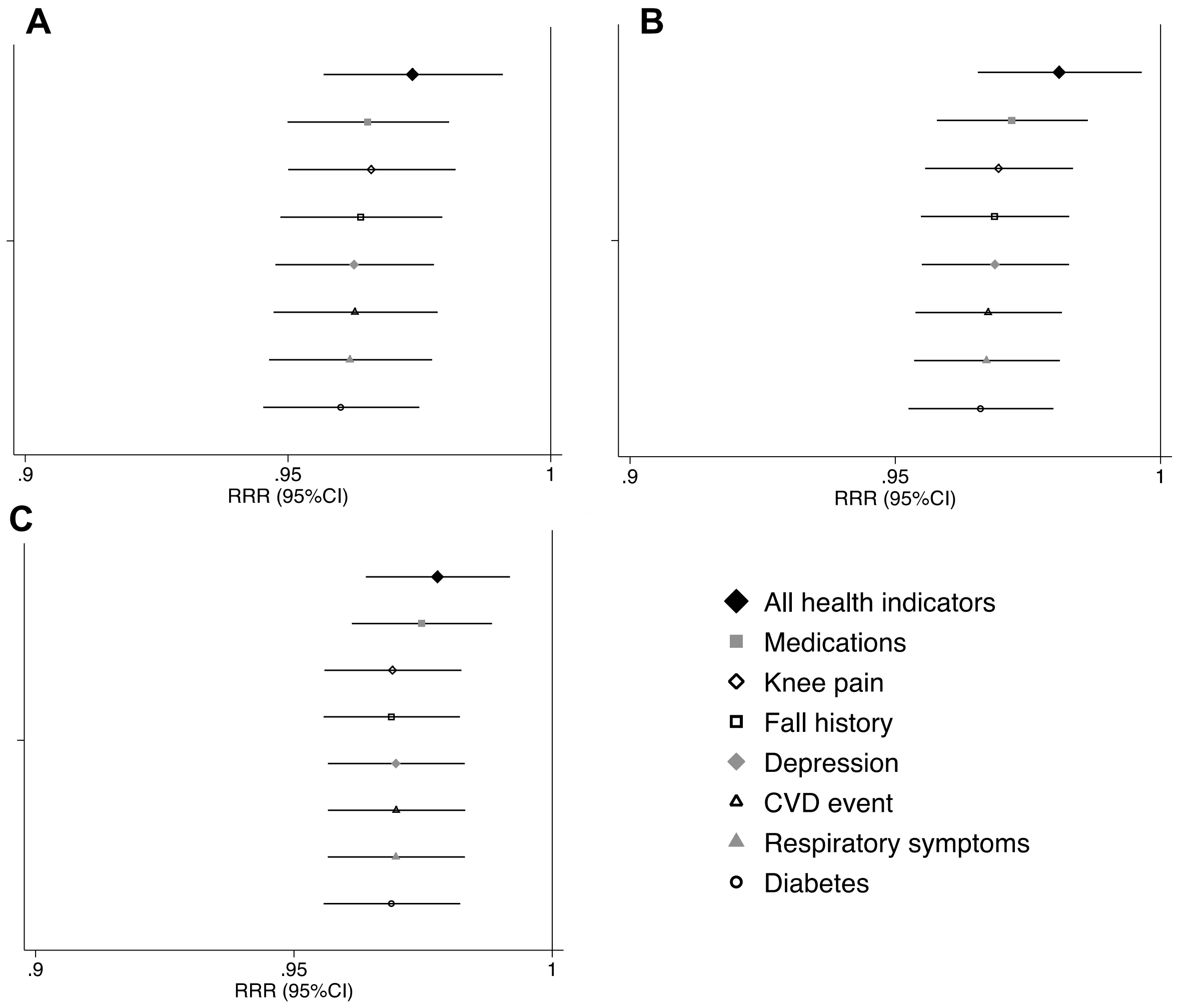

Appendix Figure 2. Relative risk ratios per 1s increase in balance with eyes open at age A. 53 and recurrent falls at age 60-64 (n=1793); B. age 53 and recurrent falls at age 68 (n=1855); C. 60-64 and recurrent falls at age 68 (n=1420) **in complete cases only**

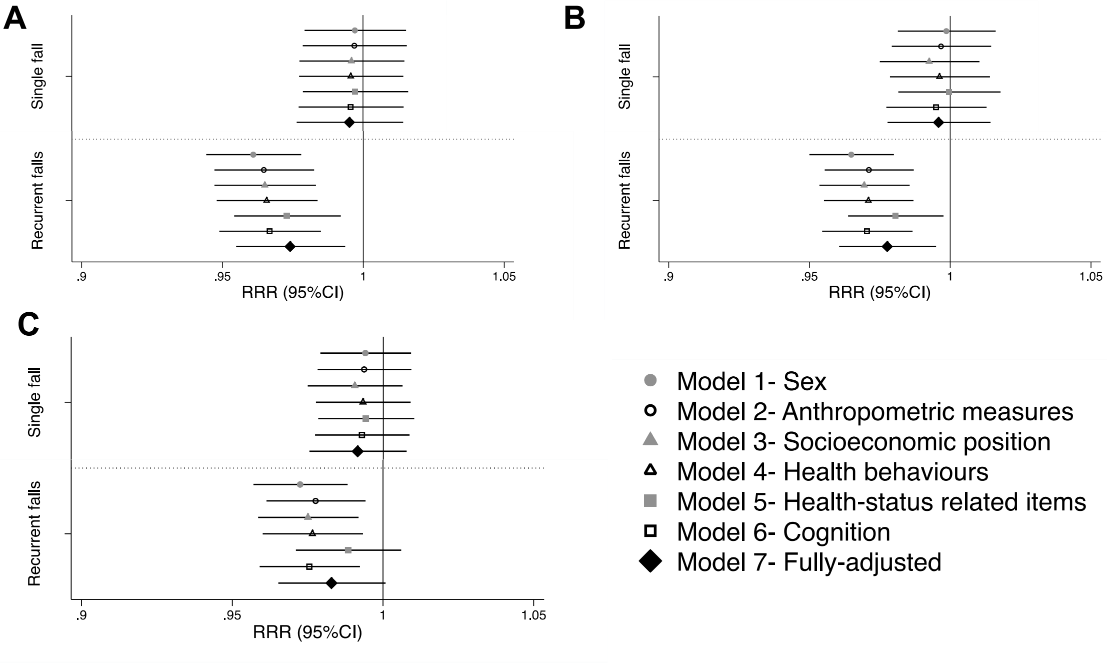

Appendix Figure 3. Relative risk ratios per 1s increase in balance with eyes open at age A. 53 and falls at age 60-64 (n=2061); B. 53 and falls at age 68 (n=2123); C. 60-64 and falls at age 68 (n=1866**) excluding those unable to perform the balance test due to health reasons who were assigned a time of 0s in main analyses**

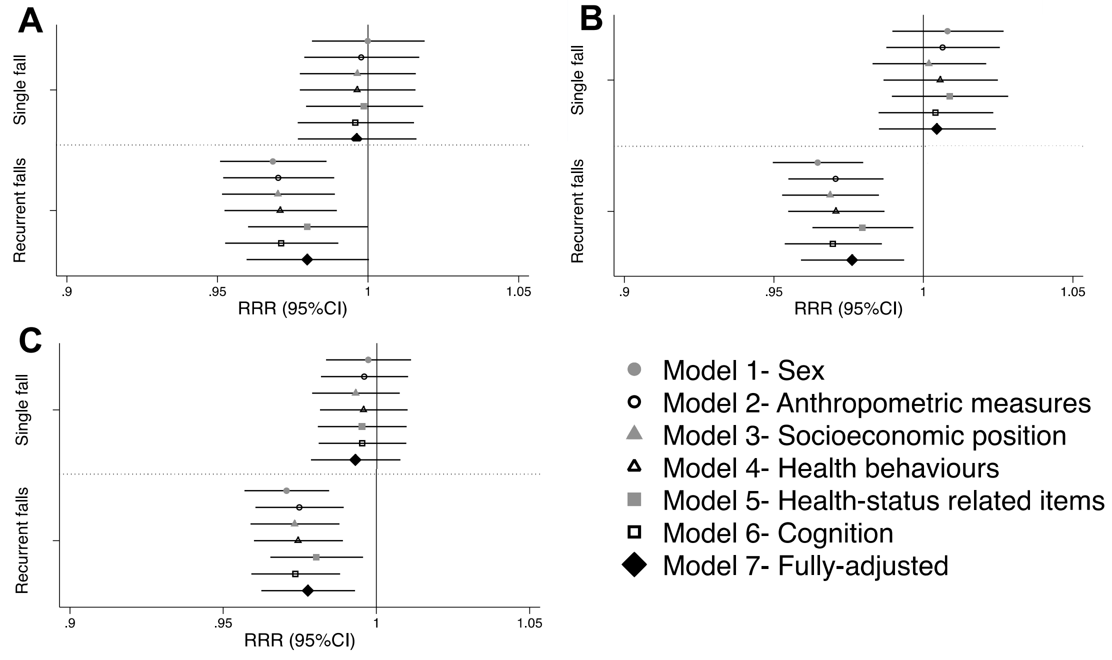

Appendix Figure 4. Relative risk ratios per 1s increase in balance with eyes open at age A. 53 and falls at age 60-64 (n=1975); B. 53 and falls at age 68 (n=2029); C. 60-64 and falls at age 68 (n=1549**) excluding those with a history of falling within the previous 12 months of balance assessment**

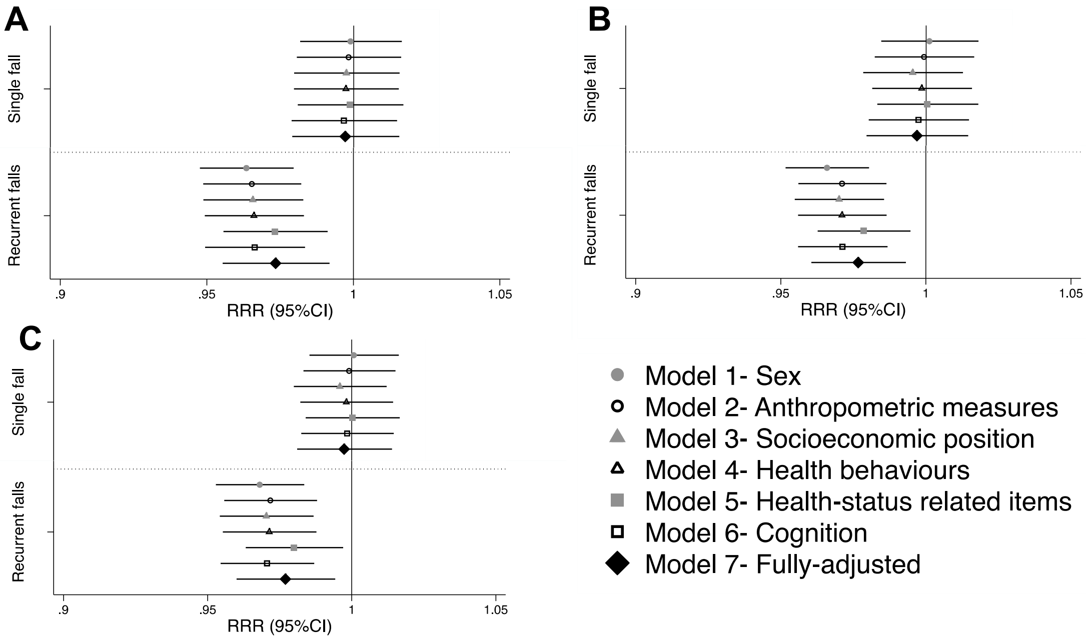

Appendix Table 1. Relative risk ratio indicating the association between change in balance (eyes open) from age 53 to 60-64 and single falls[a] at age 68

Group	Defined	Sex-adjusted model	Fully-adjusted
Stable high (n=821; 44.9%)	Achieved 30s at ages 53 and 60-64	*Reference group*	*Reference group*
Improved (n=157; 8.6%)	Improved from 0-14.99s to 15-30s or from 15-29.99s to 30s	0.73 (0.40, 1.31) p= 0.29	0.79 (0.43, 1.44) p= 0.44
Declined (n=638; 34.9%)	Declined from 30s to 0-29.99s or from 15-29.99s to 0-14.99s	1.00 (0.73, 1.38) p=0.98	1.07 (0.76, 1.48) p=0.71
Stable middle (n=70; 3.8%)	Recorded 15-29.99s at both ages	0.87 (0.39, 1.99) p= 0.75	1.00 (0.43, 2.33) p= 0.99
Stable low (n=141; 7.7%)	Stayed in 0-14.99s group	1.09 (0.63, 1.92) p= 0.76	1.22 (0.67, 2.21) p= 0.52

[a]Estimates from multinomial logistic regression presented in Table 2

[Estimates greater than 1 suggest an increased risk, while estimates less than 1 suggest a reduced risk; statistical significance is indicated if 95% Confidence intervals do not cross 1.]
